# Correlation analysis between the amniotic fluid contamination and clinical grading of neonatal hypoxic–ischemic encephalopathy and biomarkers of brain damage

**DOI:** 10.1186/s12887-024-04663-9

**Published:** 2024-03-13

**Authors:** Hongyan Lv, Fang Liu, Qiuli Wang, Zhiyong Dong, Huiming Zhang, Pengshun Ren, Liangxiang Li

**Affiliations:** 1 Department of Neonatology, Handan Maternal and Child Health Care Hospital, Handan, 056001 PR China; 2 Department of Pediatrics, NICU the 980th Hospital of the People’s Liberation Army Joint Service Support Force (Bethune International Peace Hospital), Shijiazhuang, 050082 PR China; 3 Department of Neonatal Pathology, Handan Maternal and Child Health Care Hospital, Handan, 056001 PR China; 4 Department of Neonatology and Neonatal Pathology, Handan Maternal and Child Health Care Hospital, No. 50, Li Ming Street, Hanshan District, Handan City, Hebei Province 056001 China

**Keywords:** Amniotic fluid contamination, Meconium-stained amniotic fluid, Neonatal neonatal hypoxic-ischemic encephalopathy, Serum, Tau protein, S100B, Risk factor

## Abstract

**Background:**

Amniotic fluid contamination (AFC) is a risk factor for neonatal hypoxic ischemic encephalopathy (HIE); however, the correlation between AFC level and the incidence and clinical grading of HIE, in addition to relevant biomarkers of brain damage, have not been assessed.

**Methods:**

This single-center observational study included 75 neonates with moderate-to-severe HIE. The neonates with HIE were divided into four subgroups according to the AFC level: normal amniotic fluid with HIE group (NAF-HIE), I°AFC with HIE group (I°AFC-HIE), II°AFC with HIE group (II°AFC-HIE), and III°AFC with HIE group (III°AFC-HIE). The control groups consisted of 35 healthy neonates. The clinical grading of neonatal HIE was performed according to the criteria of Sarnat and Sarnat. Serum tau protein and S100B were detected by enzyme-linked immunosorbent assay kits. Correlations of serum tau protein and S100B were evaluated using the Pearson correlation analysis.

**Results:**

(1) The incidence of neonatal HIE in the NAF-HIE group was 20 cases (26. 7%), I°AFC-HIE was 13 cases (17.3%), II°AFC-HIE was 10 cases (13.3%), and III°AFC-HIE was 32 cases (42. 7%). The incidence of moderate-to-severe HIE in the I°–III°AFC-HIE groups was 73.3% (55/75). (2) In 44 cases with severe HIE, 26 cases (59.1%) occurred in the III°AFC-HIE group, which had a significantly higher incidence of severe HIE than moderate HIE (*p* < 0.05). In NAF-HIE and I°AFC-HIE groups, the incidence of moderate HIE was 45.2% and 29.0%, respectively, which was higher than that of severe HIE (X^2^ = 9.2425, *p* < 0.05; X^2^ = 5.0472, *p* < 0.05, respectively). (3) Serum tau protein and S100B levels in the HIE groups were significantly higher than in the control group (all *p* < 0.05), and were significantly higher in the III°AFC-HIE group than in the NAF-HIE and I°AFC-HIE groups (all *p* < 0.05). (4) Serum tau protein and S100B levels in the severe HIE group were significantly higher in the moderate HIE group (all *p* < 0.05). (5) Serum tau protein and S100B levels were significantly positively correlated (*r* = 0.7703, *p* < 0.0001).

**Conclusion:**

Among children with severe HIE, the incidence of III°AFC was higher, and the levels of serum tau protein and S100B were increased. AFC level might be associated with HIE grading.

## Background

Neonatal hypoxic–ischemic encephalopathy (HIE) is not uncommon, often resulting in neonatal mortality and permanent neurological disabilities (brain palsy, epilepsy, mental disorders, etc.). In developed countries, the incidence of neonatal HIE is reported to be 1–2/1000 live births [1, 2]. In some economically poor countries, its incidence is higher, at 10–20/1000 live births [3]. There are many risk factors leading to neonatal HIE, with more than 20 risk factors currently reported, which include both maternal and fetal factors [4–7]. Nevertheless, these risk factors for HIE remain controversial.

Most scholars consider that meconium-stained amniotic fluid (MSAF) is an important factor related to newborn adverse outcomes [6, 7]. Amniotic fluid is very important for fetal growth and development. Many factors can alter the characteristics of amniotic fluid, leading to amniotic fluid contamination (AFC). The incidence of AFC in neonatal HIE was reported to be 47.3% [5], and the incidence of MSAF in neonatal asphyxia was 11.5–56.1% [8, 9]. To date, there are few reports on the assessment of different AFC levels and the incidence of HIE, clinical grading of HIE, and biomarkers of brain damage.

Tau protein is a neuronal scaffolding protein, which functions to promote microtubule assembly and stabilization [10]. S100B, secreted by astrocytes, is a calcium sensor protein, which functions to regulate the biological activities of calcium ions and is associated with cellular apoptosis and necrosis [11, 12]. Upon damage to brain tissue, the levels of tau protein and S100B in the cerebrospinal fluid and blood circulation can act as biomarkers of brain injury [13–16].

Recently, the serum concentration of tau protein and S100B have been reported to be increased in neonatal HIE (or bilirubin encephalopathy), and are suggested as biomarkers of neonatal brain damage [17–19]. This study aimed to further investigate the relationship between the AFC level and incidence of HIE, clinical grading of neonatal HIE, and biomarkers of brain damage, in a cohort of 75 neonates with HIE.

## Materials and methods

### Subjects and grouping

The single-center observational study included 75 neonates with moderate-to-severe HIE (HIE group) who were admitted to the neonatal intensive care unit of the Handan Maternal and Child Health Care Hospital from August 2018 to August 2022. The diagnostic criteria for neonatal HIE were based on the detailed content created by the Chinese Medical Association [20]. The 75 cases included 40 males and 35 females; and 31 cases with moderate HIE and 44 cases with severe HIE. All neonates were inborn. The control group consisted of 35 healthy neonates.

#### Inclusion criteria of HIE cases

(1) neonatal HIE diagnosed according to the Chinese Medical Association [20]; (2) abnormal electroencephalograms (moderate abnormalities: predominant or transient discontinuous activity; severe abnormalities: inactive (background activity<5µV) or permanent discontinuous activity (“suppression–burst” or “permanent discontinuous activity plus theta activity”) [21], paroxysmal activity and seizure patterns [22] and the diagnosis of HIE confirmed by cranial computed tomography (diffuse hypodensity in both cerebellar hemispheres and areas of the cerebral cortex, as well as abnormalities in the subcortical white matter and basal ganglia [23]) and magnetic resonance imaging (score 1: abnormal signals in the basal ganglia/thalamus, score 2: abnormal signals in the cortex, score 3: abnormal signals in the areas of cortex and basal nuclei, score 4: abnormal signals in the entire cortex and basal nuclei [22, 24]); and (3) all clinical data were intact.

#### Exclusion criteria of HIE cases

(1) intracranial hemorrhage; (2) inherited metabolic diseases; (3) congenital deformity and infectious diseases; (4) severe anemia (hemoglobin level < 120 g/L); (5) history of maternal drug abuse; and (6) patients who automatically abandon treatment. The clinical features of neonates with their mothers are shown in Table 1.

**Table 1:** Clinical features of neonates and their mothers

	Control group (*n* = 35)	HIE group (*n* = 75)	*P* value
**Maternal**			
Age, year (mean ± SD)	27.1 ± 2.9	27.1 ± 3.3	>0.05
Mode of delivery, n (%)			
Cesarean	13 (37.1)	42(56.0)	<0.05
Spontaneous vaginal delivery	20 (57.1)	27 (36.0)	>0.05
Perineotomy	2 (5.7)	6 (8.0)	<0.05
Complication of pregnancy, n (%)			
Hypertension of pregnancy	3 (8.6)	5 (6.7)	<0.05
Diabetes	1 (2.9)	4 (5.3)	<0.05
Antepartum hemorrhage	2 (5.2)	7 (9.3)	<0.05
**Neonatal**			
Gestational age, week (mean ± SD)	39.2 ± 1.3	39.1 ± 1.5	>0.05
Gender, n (%)			
Male	21 (60.0)	40 (53.3)	<0.05
Female	14 (40.0)	35 (46.7)	>0.05
body weight, g (mean ± SD)	3369.0 ± 514.4	3315.0 ± 487.2	>0.05
5 min Apgar score(mean ± SD)	8.5 ± 1.0	3.5 ± 1.4	<0.05

HIE, hypoxic–ischemic encephalopathy

Patients received a series of comprehensive supportive therapies after hospitalization, such as hypothermia [25], reduction of intracranial pressure, correction of acid–base balance disorders, anticonvulsant drugs, and so on. Patients also underwent tests to assess blood glucose levels, and liver and renal function.

This study was conducted in accordance with the Declaration of Helsinki and was approved by the Medical Ethics Committee of Handan Maternal and Child Health Care Hospital of Hebei Province. Written informed consent for the publication of the patients’ clinical details was obtained from the parents of the enrolled neonates.

### Classification of AFC degree

AFC degree was classified according to the method described in the previous study [26]. Normal amniotic fluid (non-amniotic fluid contamination) is clear. The different AFC degrees are divided into three levels: I°AFC, amniotic fluid is light green; II°AFC, amniotic fluid is yellow–green or dark-green and cloudy; and III°AFC, amniotic fluid is brown–yellow, thick, and meconium stained, also known as meconium-stained amniotic fluid (MSAF).

### **Clinical grading of neonatal HIE**

The clinical grading of neonatal HIE was estimated by Sarnat and Sarnat’s scoring methods [27].

### Determination of serum tau protein and S100B

A blood sample was taken from the vein within the first 24 h of postnatal life, which was placed in a test tube (5 ml, No. A06277602, Hebei City Zhong Xing Medical Supplies Co. Ltd, China) and kept at room temperature for 30 min, followed by centrifugation (table low speed automatic balancing centrifuge L-400 (Hunan Xiangyi Laboratory Instrument Development Co. Ltd., China)) for 15 min (3000 rpm). The serum was loaded into a test tube and stored at -70 °C. To avoid any influence on the detection results, the neonates did not undergo a blood transfusion before the collection of blood samples. The storage time from blood collection to measurement was 3 months.

Serum tau protein and S100B were detected by enzyme-linked immunosorbent assay (ELISA) kits (Shanghai HuDing Biological Science and Technology Co., Ltd. [R&D Systems, USA]; the purchased kits were Human Tau protein (T-Tau) ELISA kit and Human S-100B protein (S-100B) ELISA kit). Before the assay, the samples were removed from the − 70 °C freezer and placed on an oscillator (FWZ-1 type micro drug device, Guangzhou Fenghua Bioengineering Co. Ltd, China) at room temperature with shock melting for 1 h (1200 rpm). The ELISAs were performed following the manufacturer’s instructions.

### Statistical analysis

All data analysis was performed by SPSS 11.5 software (Chicago, IL). Measurement data were described as the mean with standard deviation (SD), and a comparison of two groups was evaluated using the Student’s t-test. Comparisons of multiple groups were performed with the Student-Newman-Keuls test. Counting data were expressed as numbers (%), the comparisons between groups were calculated by the Chi-square (X^2^) test or Fisher’s test, as appropriate. Correlations of serum tau protein and S100B were tested using Pearson correlation analysis. *p* < 0.05 was considered to indicate statistical significance.

## Results

### Incidence of neonatal HIE in different AFC groups

In the 75 patients with HIE, the incidence of neonatal HIE in NAF-HIE group was 20 cases (26.7%), I°AFC-HIE was 13 cases (17.3%), II°AFC-HIE was 10 cases (13.3%), and III°AFC-HIE was 32 cases (42.7%).The incidence of neonatal HIE in I°–III°AFC-HIE groups was 55 cases (73.3%). The incidence of severe HIE in the III°AFC-HIE group was significantly higher than that of moderate HIE (*p* < 0.05). The incidence of moderate HIE in the NAF-HIE group and I°AFC-HIE group was higher than that of severe HIE (all *p* < 0.05; Table 2).

**Table 2:** The incidence of neonatal HIE in different AFC groups n (%)

Group	Control group (*n* = 35)	Moderate HIE (*n* = 31)	Severe HIE (*n* = 44)	X^2^ value	*P* value
Normal AF	26 (74.3)	14 (45.2)	6 (13.6)^ab^	9.243	0.002
I° AFC	5 (14.3)	9 (29.0)	4 (9.1)^ab^	5.047	0.025
II° AFC	2 (5.7)	2 (6.5)	8 (18.2)^ab^	1.269	0.260
III° AFC	2 (5.7)	6 (19.4)	26 (59.1)^ab^	11.739	0.001

Note: ^a^ Compared with moderate HIE group, *p* < 0.05; ^b^ Compared with control group, *p* < 0.05

AF, amniotic fluid; AFC, amniotic fluid contamination; HIE, hypoxic–ischemic encephalopathy

### Comparison of serum tau and S100B levels in different HIE groups

The serum level of tau protein and S100B in the HIE group was significantly higher than that of control group (all *p* < 0.05). Furthermore, the tau protein and S100B level in severe neonatal HIE were significantly higher than that of moderate HIE (all *p* < 0.05; Table 3).

**Table 3:** **Comparison of serum tau and S100B levels in different HIE groups (mean ± SD)**

Group	Control group (*n* = 35)	Moderate HIE (*n* = 31)	Severe HIE (*n* = 44)	*F* value	*P* value
Tau (pg/ml)	106.4 ± 19.0	668.9 ± 150.8^a^	962.0 ± 1666.6^ab^	409.0	< 0.001
S100B (ng/L)	145.2 ± 29.0	340.3 ± 104.1^a^	588.9 ± 123.0^ab^	207.5	< 0.001

Note: ^a^ Compared with control group, *p* < 0.05; ^b^ Compared with moderate HIE, *p* < 0.05

HIE, hypoxic–ischemic encephalopathy

### Comparison of serum tau protein and S100B level in different AFC groups

The serum level of tau protein and S100B in the NAF-HIE group, I°AFC-HIE group, II°AFC-HIE group, and III°AFC-HIE group were significantly higher than that of control group (all *p* < 0.05). The serum tau protein and S100B level in the III°AFC-HIE group were significantly higher than those of the NAF-HIE group and I°AFC-HIE group (all p<,0.05); however, the tau protein and S100B level in the III°AFC-HIE group showed no significant difference to that of the II°AFC-HIE group (all *p* > 0.05; Table 4).

**Table 4:** Comparison of serum tau protein and S100B levels in different AFC groups (mean ± SD)

Group	*n*	Tau (pg/ml)	S100B (ng/L )
Control group	35	106.4 ± 19.0	145.2 ± 29.0
NAF-HIE	20	725.1 ± 247.9	385.7 ± 167.2 ^a^
I°AFC-HIE	13	725.1 ± 133.1^a^	411.7 ± 125 0.8 ^a^
II°AFC-HIE	10	849.4 ± 166.1^a^	460.1 ± 110.0 ^a^
III°AFC-HIE	32	956.5 ± 175.0^abc^	587.3 ± 146.2^abcd^
*F* value		32.700	7.140
*P* value		< 0.001	< 0.001

Note: ^a^ Compared with control group, *p* < 0.05; ^b^ Compared with NAF-HIE, *p* < 0.05

^c^ Compared with I°AFC-HIE, *p* < 0.05; ^d^ Compared with II°AFC-HIE, *p* < 0.05

AF, amniotic fluid; AFC, amniotic fluid contamination; HIE, hypoxic–ischemic encephalopathy

### Correlation analysis of serum tau protein and S100B level

**Fig. 1:**
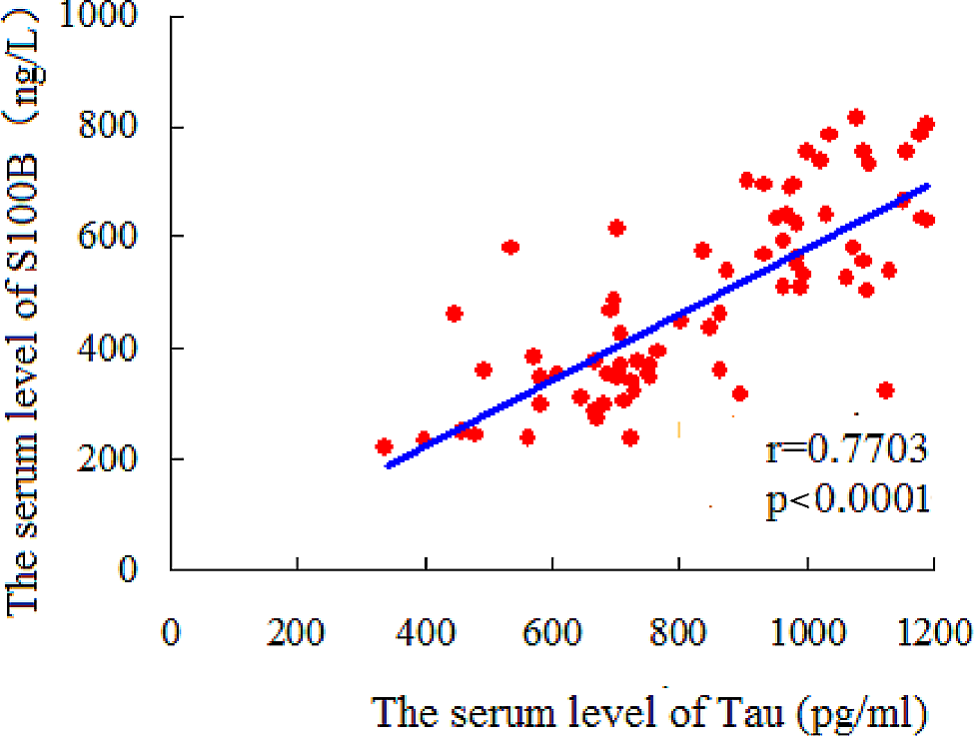
Correlation between serum tau protein and S100B levels

Our results showed that the levels of serum tau protein and S100B were significantly positively correlated (*r* = 0.7703, *p* < 0.0001; Fig. 1).

## Discussion

Neonatal HIE is characterized by brain injury due to severe ischemia–hypoxia of the cerebrum during the prenatal and perinatal period [28]. A histopathological study has identified that neonatal HIE involves neuronal degeneration and necrosis, periventricular leukomalacia, intracranial hemorrhage, and various pathological changes. According to relevant report on neonates with hypoxic brain injury, approximately 80% of cases occur in the prenatal period and 10–20% of cases occur in the perinatal period [29]. Many risk factors are associated with neonatal HIE, such as AFC [4, 5, 30–34], low birth weight (< 2.5 kg) [33], gestation age ≥ 41 weeks, umbilical cord around neck [35], newborn born to women without reproductive history [5], placental abruption, ruptured uterus [31], placenta previa, dystocia, fetal respiratory distress syndrome, emergency cesarean Sects. [4, 36], growth retardation, large head circumference [30], chorioamnionitis [36], Apgar score [31], and so on. Nevertheless, some inconsistent results remain among these risk factors for HIE.

Normally, the amniotic fluid, which is the internal environment of the growing fetus, is a colorless and transparent liquid. AFC was reported to be an independent risk factor for neonatal HIE [31, 33]; especially, MSAF showed a significant correlation with the occurrence of neonatal HIE [32]. The causal chain of events resulting in AFC and neonatal HIE, influence of the AFC level on the incidence and severity of HIE, as well as the biomarkers of brain damage are not yet fully understood. Research into the mechanisms has gained increasing attention recently.

The clinical grading of neonatal HIE is divided into three levels: mild, moderate, and severe HIE. The AFC level can be divided into three levels: I°AFC, II°AFC, and III°AFC (also known as MSAF). There have been many reports regarding AFC in neonatal asphyxia; the incidence of AFC in neonatal asphyxia was 11.5–56.1% [8, 9], and severe neonatal asphyxia may be involved in multiple organ damage, such as neonatal HIE, respiratory distress syndrome, neonatal necrotizing enterocolitis, renal and liver damage. Among these adverse consequences, neonatal HIE was of the greatest concern to obstetricians and neonatologists. The incidence of AFC causing neonatal HIE has been reported in few studies; the incidence of AFC causing neonatal HIE was 47.3–51.2% [4, 5]. Our study showed that the incidence of neonatal HIE (moderate-to-severe HIE cases) in the I°–III° AFC groups was 73.3% (55/75). This result was significantly higher than the results of Chen et al. (X^2^ = 12.4198, *p* = 0.004) and Wang et al. (X^2^ = 70.1199, *p* = 0.0000) [4, 5].

III°AFC is also known as MSAF. MSAF is one of the clinical manifestations of the fetus in intrauterine hypoxia. A clinical epidemiological study reported that the incidence of MSAF was 16.6% [37]. MSAF is the most harmful for newborns, and the incidence probability of MSAF in neonatal HIE was higher. Li et al. reported that the incidence of MSAF in neonatal HIE was 49.6% [38]. Torbenson et al. reported that the incidence of MSAF in neonatal HIE was 42.3% [32]. Our data showed that the incidence of neonatal HIE (including moderate-to-severe HIE cases) in the III°AFC (or MSAF) group was 42.7% (32/75); the result was in keeping with those of previous studies. Therefore, we also confirmed that MSAF was a risk factor for neonatal HIE, and that it had an important reference value for judging the severity of neonatal HIE.

Regarding the relationship between the AFC level and HIE clinical grading, our result showed that in the III°AFC-HIE group, the incidence of severe HIE was 59.1% (26/44), which was significantly higher than that of moderate HIE (19.4%, *p* < 0.05). In the NAF-HIE group, the incidence of moderate HIE was 45.2% (14/31), which was significantly higher than that of severe HIE (13.6%, *p* < 0.05). In the I°AFC-HIE group, the incidence of moderate HIE was 29% (9/31), which was higher than that of severe HIE (9.1%, *p*<0.05). These results suggested that the higher the AFC level, the higher the probability of neonatal severe HIE.

When no contamination is present in the amniotic fluid, it is a clear liquid, which is very important to fetal growth and development. If the fetus suffers from intrauterine hypoxia, leading to an enhanced intestinal peristalsis, relaxation of the anal sphincter occurs allowing meconium into the amniotic fluid. As a result, the fetus inhales the contaminated amniotic fluid, causing respiratory tract obstruction and further aggravating the hypoxic–ischemic brain damage. Therefore, amniotic fluid monitoring during pregnancy should be routinely performed.

Of note, in normal amniotic fluid, neonatal HIE can also occur. Recently, Wang JR et al. reported that the incidence of moderate-to-severe HIE in patients with no amniotic fluid contamination was 48.8% [4]. We also observed this in the present study; in the NAF-HIE group (non-amniotic fluid contamination group), the incidence of moderate-to-severe HIE was 26.7% (20/75). This result was lower than that of Wang JR et al. Because a certain number of neonatal HIE cases occurred in the normal amniotic fluid group, this suggests that the risk factors for neonatal HIE are more complex, which requires further study.

To reveal the relationship between the AFC level and severity of neonatal HIE, in addition to biomarkers of brain damage, we performed this preliminary study. Tau protein, a microtubule-associated structural protein family member, it is located in the axons and dendrites of central neurons [39–41]. Tau promotes the formation and stability of microtubules, and regulates the growth and development of neurons as well as the communication of axons [42]. S100B is a calcium sensor protein, and a multifunctional member of S100-calmodulin-troponin super-family. S100B functions to regulate the growth and differentiation of cells. In vitro and in vivo experiments showed that S100B may stimulate the proliferation of glial cell [43, 44], and it was considered to be a brain-specific protein. Previous studies have shown that tau protein and S100B have significant value in estimating brain damage, for instance, brain trauma [45–49], ischemic stroke [14, 50, 51], and cerebral hemorrhage [52, 53]. In recent years, tau protein and S100B have also been reported to be associated with newborn hypoxia and newborn brain injury, including neonatal jaundice encephalopathy [17], neonatal asphyxia [54–56], and neonatal HIE [18, 56–58]. Our results demonstrate that serum tau and S100B levels of neonates with moderate-to-severe HIE were significantly higher than in the control group. Furthermore, serum tau protein and S100B levels in neonates with severe HIE were significantly higher than those of patients with moderate HIE, suggesting that the levels of serum tau protein and S100B could serve as biomarkers of neonatal brain damage. We showed that the serum level of tau protein and S100B in all HIE groups were significantly higher than in the control group. Furthermore, serum tau and S100B levels in the III°AFC-HIE group were significantly higher than in the NAF-HIE and I°AFC-HIE groups, which is likely related to the higher incidence of severe HIE cases in the III°AFC-HIE group, and the higher incidence of moderate HIE cases in the NAF-HIE and I°AFC-HIE groups; therefore, the higher the AFC level, the more serious the neonatal brain damage.

Serum tau protein and S100B represent the number of neuronal and glial cells, respectively; therefore, their levels in the serum reflect the degree of damage to neuronal and glial cells. In this study, serum tau protein and S100B levels were significantly positively correlated (*r* = 0.7703, *p* = 0.0001), suggesting that neurons and glial cells in neonatal HIE were affected to a similar degree. How glial cells can be repaired or regenerated, in addition to the functional reconstruction of neurons, in neonatal HIE still needs to be further studied.

However, the study has some limitation. First, this is a retrospective study, and inherent limitations exist in this kind of studies. Second, the grading of AFC relies solely on color, this method is subjective.Factors such as individual differences, variations in color perception, and the influence of lighting conditions can contribute to this subjectivity. Further studies are needed to explore more objective and standardized methods for AFC grading.

In conclusion, our findings showed that among children with severe HIE, the incidence of III°AFC was higher, and the levels of serum tau protein and S100B were increased, suggesting that AFC level might be associated with HIE grading.

## Data Availability

The datasets used and/or analysed during the current study are available from the corresponding author on reasonable request.

## Abbreviations

AFC: amniotic fluid contamination
HIE: hypoxic ischemic encephalopathy
NAF: normal amniotic fluid
MSAF: meconium stained amniotic fluid
